# Gleanings from the Minutes of the Medical Society of the City of Wheeling, and County of Ohio, West Virginia

**Published:** 1872-05

**Authors:** W. J. Bates

**Affiliations:** President


### GLEANINGS FROM THE MINUTES OF THE MEDICAL SOCIETY OF THE CITY OF WHEELING, AND COUNTY OF OHIO, WEST VIRGINIA.

DR. W. J. BATES, *President.* REPORTED BY DR. S. L. JEPSON, *Secretary.*

DR. HILDRETH.—I was recently called to a boy who had injured his tongue severely, by a fall, it being caught between the teeth. I found the tongue cut one-third or one-half through laterally. After some difficulty, I succeeded in introducing a threaded needle from behind forward, and brought the inside edges as nearly as possible in apposition. A good result followed. I remember a similar case which occurred here some years ago, and Doctor —— utterly failed, after repeated efforts, to introduce a suture. He abandoned the case to nature. I think that in most cases the ordinary vulsellum forceps will suffice to hold the tongue, while the stitch is being placed.

DR. FRISSELL.—I have seen several tongues injured in this way, one very bad case. Remember seeing Mutter operate, some years ago, for this injury, using a hook to keep the tongue in proper position. I have since resorted to the same means, and consider it the best.

DR. R. H. CUMMINS.—I have seen several injured tongues in my early practice, and experience has taught me that the wound does better when left to nature, unless the injury be very severe, such as the case first related. I think that Bushe's hemorrhoidal needle would serve an admirable purpose in these cases, its peculiar shape adapting it well to the introduction of a suture.

DR. FRISSELL.—While on this subject, I wish to mention a case of some interest to me, that came under my care a few years ago. The patient was a soldier, who had received a gun-shot wound of the mouth, by which some of the teeth were knocked out, and the tongue badly injured. He was treated, and all wounds healed, except that of the tongue. A small opening remained in the side of the tongue, from which pus was occasionally discharged; the organ, at such times, becoming inflamed, and giving considerable annoyance. When the case was brought to me, I suspected the presence of some foreign body, and making an incision of sufficient size, I found and removed the crown of a molar tooth, which had lain deeply imbedded in the tongue for years. Complete recovery ensued.

Dr. Bates.—A few weeks ago, on Sunday night, I delivered a patient of a healthy child. Meconium passed from the child's bowels naturally that night and the next day. On Monday night, however, the stools became quite bloody, and until Wednesday, sixteen stools, all bloody, were passed; the blood of the first being somewhat clotted, but afterwards thinner. After Wednesday, the operations became gradually natural. Gallic acid was used. I believe this is the first case of the kind I have ever seen.

One of those annoying cases of obstinate constipation, the result of want of care during pregnancy, fell to my lot recently. I delivered the woman—who was rather robust and somewhat inclined to obesity—of a healthy child. Bowels had previously moved daily. Several days after confinement, the patient objecting to oil, and the bowels not being opened, an enema was given, and satisfactory results obtained. There was considerable milk-fever, which, however, soon subsided. Bowels continued costive for several days. About the tenth day, severe pain set in, and there was a great desire to go to stool, but any effort to evacuate the bowels was accompanied with severe tormina and tenesmus. Painful hemorrhoids appeared. In examining them, I accidentally ruptured one, and some bleeding followed, which gave partial and temporary relief. A full anodyne, rendered necessary by the excessive pain, was given, and relief of some hours' duration followed. The pain returned in the night, with great severity, and I was called. An ounce of castor oil was given, but without effect; also, citrate and sulphate of magnesia and other cathartics; but still to no purpose. A rectal examination, with a view to break up and remove hardened fæces, was stoutly resisted by the patient. Pills of calomel, blue-mass and colocynth, oil, salts and enemata were all again tried, but in vain. The pain became more violent, and finally, after many hours of suffering, scybala began to pass. Finally, the pain became equal in severity to labor-throes, and the labor ended by the expulsion, with a loud thug, of a scybalous mass as large as a man's two fists, and this was followed by a large quantity of semi-fluid fæces. Paralysis of the sphincter ani resulted, lasting for two days, when it began to regain its power, which was soon completely restored. Here was a case in which the bowels were said to be regular during the whole of pregnancy, and yet it is evident, from the exceedingly annoying and painful termination, that they were really costive; that is, the operations were not of sufficient frequency or size to carry off the accumulating waste material. It is a matter of no small importance, and a point which this case should impress, to keep the bowels of the pregnant woman in a soluble condition.

Dr. Jepson.—A similar case, though of less severity, and occurring in a man, came under my care recently. The patient was an intemperate man, much addicted to drinking hard cider, which he said he drank as a substitute for liquor. He was of costive habits, though his bowels moved daily. Excessive pain came on, and purgative pills were prescribed, as also enemata. When I first saw him, he had used both to no purpose. He had "taken a whole box of pills prescribed by Dr. ——, and would have been taking them yet had they not run out." The pain was at times severe, and the patient somewhat exhausted. I first ordered copious enemata of water, with salt or soap. In the evening these had produced no effect. I then ordered ʒ ij. castor oil every hour and a half, or two hours. The patient and friends were exceedingly doubtful as to these little doses accomplishing anything, since he had failed with three or four times this dose. However, they faithfully carried out directions, and the next morning I met the patient with his countenance wreathed in smiles, for complete success had followed the use of these small doses of oil, and his bowels had been emptied of "almost a chamber-full" of very offensive fæces. Complete subsidence of pains, and restoration to perfect health followed.

These cases may be regarded as simple, but they are sometimes exceedingly troublesome, and being not infrequent, a proper treatment becomes an important consideration. That small doses of castor oil, frequently repeated, have very often an excellent effect, I know from trial, in other cases than the one cited. Doubtless other cathartics, given in the same way, are more efficient than when given in one large dose, but I am inclined to credit the oil with some peculiar virtue. At any rate, it is worthy of a trial, and so long as it serves me so well, I will continue to use it.

Another mode of treatment that I have seen result in success, in cases of obstinate constipation, where everything else failed, is the external application of croton oil and castor oil combined. About four to eight drops of the former are mixed with sufficient castor oil to prevent severe vesication, and then thoroughly rubbed in, near the umbilicus. The first time I ever used this, was by the direction of Dr. White, while I was a resident physician in the Cincinnati Hospital. The patient was a young woman whose bowels had not been opened for over a week, notwithstanding everything, almost, that skill or ingenuity could suggest, had been tried. A speedy and excellent result followed this treatment. Doubtless the friction itself accomplished some good, but that the principal effect was from the oil, acting through absorption, I cannot doubt. Try it.

